# The Mechanism of Action of the Active Ingredients of *Coptidis rhizoma* against Porcine Epidemic Diarrhea Was Investigated Using Network Pharmacology and Molecular Docking Technology

**DOI:** 10.3390/v16081229

**Published:** 2024-07-31

**Authors:** Hong Zou, Zheng Niu, Zhangchen Tang, Peng Cheng, Yanling Yin, Gan Luo, Shilei Huang

**Affiliations:** 1 College of Animal Science & Technology, Chongqing Three Gouges Vocational College, Chongqing 404100, China; hong-zou@outlook.com (H.Z.); yanlinglucky@126.com (Y.Y.); 2 College of Veterinary Medicine, Northwest A & F University, Xianyang 712000, China; nz0511@126.com; 3 Integrative Science Center of Germplasm Creation in Western China (CHONGQING) Science City, Biological Science Research Center, Southwest University, Chongqing 402460, China; tangzhangc@email.swu.edu.cn; 4 Wanzhou Center for Animal Husbandry Industry Development of Chongqing, Chongqing 404100, China; dys1234567@email.swu.edu.cn

**Keywords:** PEDV, *Coptidis rhizoma*, network pharmacology, small molecule docking

## Abstract

The objective of this study was to elucidate the mechanism of action of the active components of *Coptidis rhizoma* against porcine epidemic diarrhea and to provide a theoretical foundation for further development of novel anti-PED therapeutic agents based on *Coptidis rhizoma*. The potential targets of *Coptidis rhizoma* against PEDV were identified through a comprehensive literature review and analysis using the TCMSP pharmacological database, SwissDrugDesign database, GeneCards database, and UniProt database. Subsequently, the STRING database and Cytoscape 3.7.1 software were employed to construct a protein–protein interaction (PPI) network and screen key targets. Gene Ontology (GO) function and Kyoto Encyclopedia of Genes and Genomes (KEGG) pathway enrichment analysis were conducted on the identified targets. Molecular docking studies were performed using AutoDock 1.5.7 software to analyze the binding energy and modes of interaction between the active components of *Coptidis rhizoma* and the target proteins. The PyMOL 2.5.0a0 software was employed to visualize the docking results. Through comprehensive analysis, 74 specific targets of active components of *Coptidis rhizoma* against PEDV were identified. The core gene targets were screened, and an interaction network diagram was subsequently generated. Ultimately, 14 core targets were identified, with STAT3, ESR1, CASP3, and SRC exhibiting the most significant interactions. GO enrichment analysis revealed a total of 215 molecular items, including 48 biological function items, 139 biological process items, and 28 cellular component items. KEGG enrichment analysis identified 140 signaling pathways. Molecular docking analysis demonstrated that epiberberine and palmatine exhibited high binding affinity with STAT3 protein, worenine showed high binding affinity with ESR1 protein, obacunone exhibited high binding affinity with CASP3 protein, and epiberberine, obacunone, berberine, and berberruine exhibited high binding affinity with SRC protein. A network pharmacology and molecular docking technology approach was employed to screen six important active components of *Coptidis rhizoma* and four important potential targets against PEDV infection. The findings indicated that the active components of *Coptidis rhizoma* could serve as promising pharmaceutical agents for the prevention and control of PEDV, with significant potential for clinical application.

## 1. Introduction

Porcine epidemic diarrhea (PED) is an acute, highly contagious enteric disease characterized by diarrhea, vomiting, dehydration, and high mortality, with rates reaching up to 100% in suckling piglets within their first week of life. The disease is mainly caused by infection with the porcine epidemic diarrhea virus (PEDV), a single-stranded, positive-sense RNA virus of the Alphacoronavirus genus of the Coronaviridae family [1]. Currently, there are no effective pharmacological interventions for the prevention and control of PED. Traditional inactivated and live vaccines are considered the most effective and economical strategies for PED management. However, the emergence of PEDV mutant strains has rendered vaccine efficacy suboptimal. Furthermore, the base of pig breeding in China is large, the breeding mode is diverse, and the main body of disease prevention and control is transferred. The variability in understanding the genesis, evolution, pathogenesis, dissemination, and immunological responses of PED among various stakeholders has led to inconsistent approaches to disease management [2]. The ongoing immune and environmental pressures have driven adaptive evolution of PEDV, resulting in the continuous emergence of new strains or subtypes through genetic variation. In the same farm, there are not only PEDV, but also other diarrheal pathogens. For example, transmissible gastroenteritis virus (TGEV), porcine colibacillosis, and porcine deltacoronavirus (PDCoV) are other pathogens that can coexist and cause mixed infections in the same field. This multi-pathogen coexistence and mixed infection contributes to the current situation of PED prevention and control in China, which remains challenging [3].

*Coptidis rhizoma* is a significant component within the domain of Chinese herbal medicine. Scientific studies have demonstrated that *Coptidis rhizoma* and its active ingredients play a crucial role in protecting cardiovascular and cerebrovascular systems, exhibiting hypoglycemic, anti-inflammatory, antitumor, and antimicrobial properties, and modulating intestinal flora [4]. The existing reports indicate that berberine, the active ingredient of *Coptidis rhizoma*, can inhibit PEDV infection by regulating the host signal transduction pathway or virus life cycle. Berberine has been demonstrated to significantly inhibit PEDV infection. Its mechanism of action appears to primarily involve the replication and assembly of PEDV, although the specific molecular mechanisms remain to be further elucidated [5]. To determine whether other active ingredients of *Coptidis rhizoma* can also play a role in anti-PEDV, this study employed network pharmacology–molecular docking technology to first identify the main active ingredients of *Coptidis rhizoma* and then to identify the main interaction targets of the *Coptidis rhizoma* active ingredients against PEDV. The study then used molecular docking technology to verify the main action sites of the identified compounds, with the aim of providing assistance for the development of anti-PEDV drugs based on *Coptidis rhizoma* drugs.

## 2. Materials and Methods

In order to identify the principal chemical constituents of *Coptidis rhizoma*, the keyword “*Coptidis rhizoma*” was searched in the TCMSP pharmacology database (https://www.tcmsp-e.com) on 24 January 2024. Subsequently, the active ingredients with oral bioavailability (OB) ≥ 30% and which were drug-like (DL) ≥ 0.18 were selected to obtain the effective active ingredients of *Coptidis rhizoma* in this study. On 25 January 2024, the SwissADME tool within the SwissDrugDesign database (https://www.molecular-modelling.ch/swiss-drug-design.html) was utilized to screen the active compounds of *Coptidis rhizoma*, identifying those with superior intestinal absorption and drug-like properties. The SwissTargetPrediction tool was subsequently used to screen the effective targets of *Coptidis rhizoma* active compounds. Subsequently, the GeneCards database (https://www.genecards.org) was employed to identify disease-related gene targets associated with PED on 25 January 2024. The keyword “Porcine Epidemic Diarrhea” was utilized for retrieval on 25 January 2024. Finally, the drug targets and disease-related target genes obtained from each database were integrated and deduplicated, and their names were standardized using the UniProt database (https://www.uniprot.org) on 25 January 2024.

The disease targets of the effective active components of *Coptidis rhizoma* and PED disease targets were used to create a Venn diagram using Venny2.1 (https://bioinfogp.cnb.csic.es/tools/venny/index.html) to explore the targets of the active components of *Coptidis rhizoma* against PED disease on 26 January 2024.

The SMILES structure was constructed using the PubChem function of the NCBI database (https://www.ncbi.nlm.nih.gov) on 26 January 2024. Subsequently, the pharmacokinetics and drug-likeness of the active components of *Coptidis rhizoma* were evaluated using the SwissDrugDesign database on 26 January 2024. If the intestinal absorption capacity of pharmacokinetics is high and the drug exhibits more than two positive responses, it is deemed suitable for use.

The 69 intersection gene targets were imported into the database based on the STRING database (https://cn.string-db.org) to obtain the interaction network of intersection gene targets on 27 January 2024. Subsequently, the Cytoscape 3.7.1 software was employed to identify the principal targets of active drugs derived from *Coptidis rhizoma* that exhibit efficacy against PEDV. This was accomplished by utilizing degree centrality (degree = 7.727272727), closeness centrality (closeness = 0.005981415), and betweenness centrality (betweenness = 111.030303) to assess the interaction strength of the principal targets.

The related genes of the 69 intersection gene targets were analyzed using the DAVID database (https://david.ncifcrf.gov), an online enrichment database, on 27 January 2024. Gene Ontology (GO) and Kyoto Encyclopedia of Genes and Genomes (KEGG) pathway enrichment were conducted on the ten most closely related genes. The Benjamini–Hochberg procedure was applied to control the false discovery rate (FDR), setting a threshold of FDR < 0.05. The results of the Gene Ontology (GO) and Kyoto Encyclopedia of Genes and Genomes (KEGG) enrichments are presented in the form of a bubble diagram.

Molecular docking and binding energy calculations were performed on the key effective active ingredients (e.g., epiberberine, palmatine, worenine, obacunone, berberine, and berberruine) and key targets (e.g., STAT3, ESR1, CASP3, and SRC). Some three-dimensional model structures of pig-derived proteins, including STAT3, ESR1, CASP3, and SRC proteins, were constructed based on the previous Swiss-model website. Subsequently, AutoDock1.5.7 software was utilized to modify the protein model, including the removal of pertinent water molecules and metal ions, followed by the uploading of receptors and ligands for flexible docking. Subsequently, the binding mode of small molecule drugs and proteins was identified through a process of screening and visualization, utilizing the Pymol 2.5.0a0 software. This entailed an analysis of the binding energy and binding position in order to ascertain the optimal binding mode. High-performance Computing (Sugon, I950r-G, China) was used for data processing.

## 3. Results

### 3.1. Drug Active Ingredients and Disease Targets

**Figure 1:**
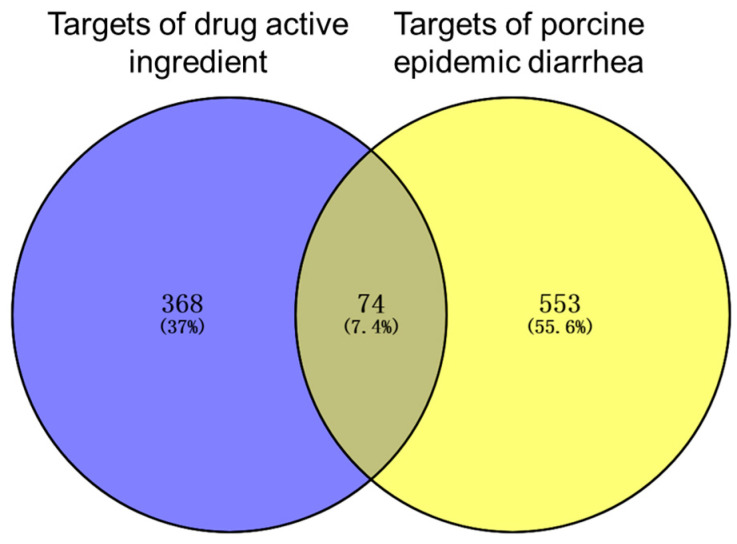
Venn diagram of intersection gene target between active components of *Coptidis rhizoma* and PED.

The active ingredients of *Coptidis rhizoma* were screened in the TCMSP database. The screening conditions were OB ≥ 30% and DL ≥ 0.18, and 14 effective active ingredients were obtained (Table 1). A total of 973 disease target genes of active ingredients of *Coptidis rhizoma* were predicted. After removing the number of repeated targets, the remaining 368 and 553 PED disease targets were identified. The online tool Venny2.1 was employed to identify the gene targets of the intersection of the active components of *Coptidis rhizoma* and PED. A total of 74 gene targets were identified, representing 7.4% of the total. Of these, 37% were attributed to the active components of *Coptidis rhizoma*, while 55.6% were attributed to PED (Figure 1).

### 3.2. The Core Gene Targets of the Effective Active Components of the Drug against PED and Their Interaction Network Diagram

**Figure 2:**
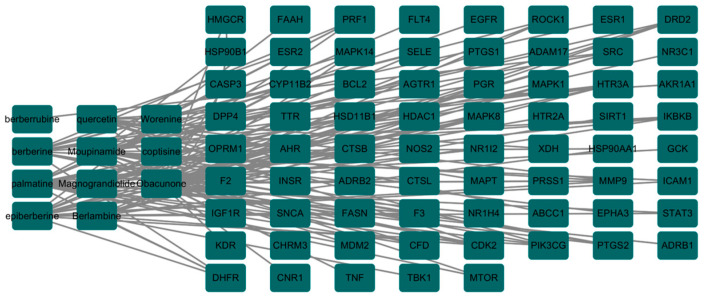
Network diagram of effective active components–intersection gene target of *Coptidis rhizoma*.

The final 11 active components of *Coptidis rhizoma* were screened using the SwissDrugDesign database, confirming that their pharmacokinetics and drug-like properties met the required criteria. The relationship between the drug targets corresponding to these 11 active components of *Coptidis rhizoma* and the 74 intersection gene targets was then examined, resulting in the identification of 69 disease gene targets. A network diagram illustrating these relationships was constructed using the Cytoscape 3.7.1 software (Figure 2).

**Figure 3:**
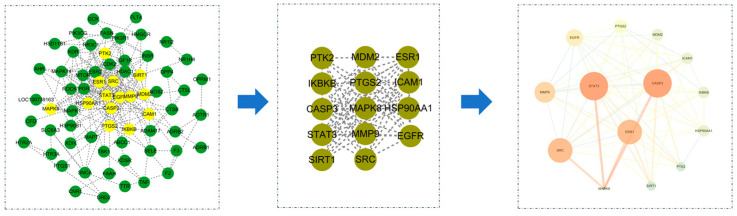
Core gene targets and interaction network of effective active components of *Coptidis rhizoma* against PED.

The STRING database was employed to identify 71 nodes and 255 edges among the core gene targets and their interactions of the effective active components of *Coptidis rhizoma* against PED. Subsequently, further screening was conducted using the Cytoscape 3.7.1 software. The screening was primarily based on closeness, betweenness, and degree values (0.005981415, 111.030303, and 7.727272727, respectively). Ultimately, 14 nodes and 65 edges were subjected to screening. The primary disease-related targets associated with pediatric epilepsy (PED) are STAT3, ESR1, CASP3, SRC, MMP9, EGFR, PTGS2, MDM2, ICAM1, IKBKB, HSP90AA1, PTK2, SIRT1, and MAPK8. Among these, STAT3, ESR1, CASP3, and SRC exhibit the greatest degree of interaction (Figure 3).

### 3.3. Enrichment Analysis of Anti-PED of Effective Active Ingredients of Drugs

**Figure 4:**
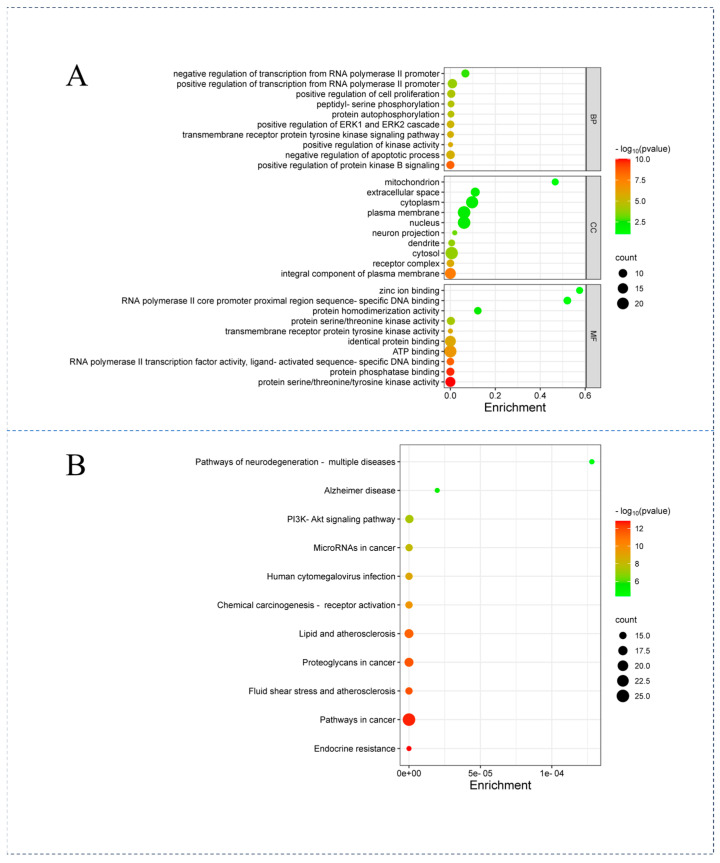
Bubble chart of active components in Coptidis Rhizoma drugs against PED. (**A**): Top 10 results of GO enrichment analysis of 215 biological functions, biological processes, and cellular components. (**B**): Top 10 results from KEGG enrichment analysis of 140 signaling pathways. Each bubble’s color represents the negative log10 value of the *p*-value, and the size of the bubble generally represents the number of genes involved in the pathway.

A total of 215 molecular items of GO enrichment analysis were obtained from the DAVID database, including 48 items of molecular function (MF), 139 items of biological process (BP), and 28 items of cellular components (CCs). The intersection of gene targets was found to be highly enriched in a number of molecular functions, including ATP binding, affinity protein binding, protein serine/threonine/tyrosine kinase activity, protein phosphatase binding, protein serine/threonine kinase activity, RNA polymerase II transcription factor activity, ligand-activated sequence-specific DNA binding, protein homologous dimerization activity, RNA polymerase II core promoter proximal region sequence-specific DNA binding, zinc ion binding, and transmembrane receptor protein tyrosine kinase activity. The biological processes that are highly enriched include positive regulation of RNA polymerase II promoter transcription, negative regulation of apoptosis process, positive regulation of protein kinase B signal, positive regulation of cell proliferation, negative regulation of RNA polymerase II promoter transcription, positive regulation of ERK1 and ERK2 cascades, transmembrane receptor protein tyrosine kinase signaling pathway, protein autophosphorylation, peptide-serine phosphorylation, and positive regulation of protein kinase activity. The cell composition is highly enriched, including plasma membrane, nucleus, cytoplasm, cytoplasm, plasma membrane integral component, extracellular space, receptor complex, dendritic cells, mitochondria, and neuron projection. A total of 140 KEGG enrichment analysis items were obtained, with a higher degree of aggregation in cancer signaling pathways, proteoglycans in cancer, lipids and atherosclerosis, the PI3K-Akt signaling pathway, fluid shear stress and atherosclerosis, chemical carcinogenesis-receptor activation, human cytomegalovirus infection, microRNA in cancer, and endocrine resistance and Alzheimer’s disease (Figure 4).

### 3.4. Molecular Docking Verification of the Main Genes Corresponding to the Active Components of the Drug and the Main Proteins Related to the Disease

**Figure 5:**
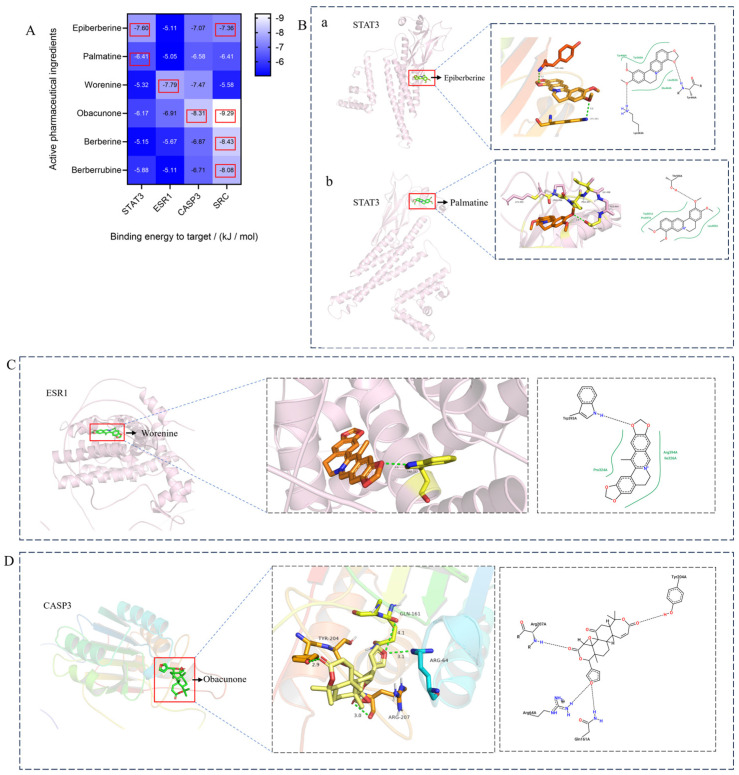

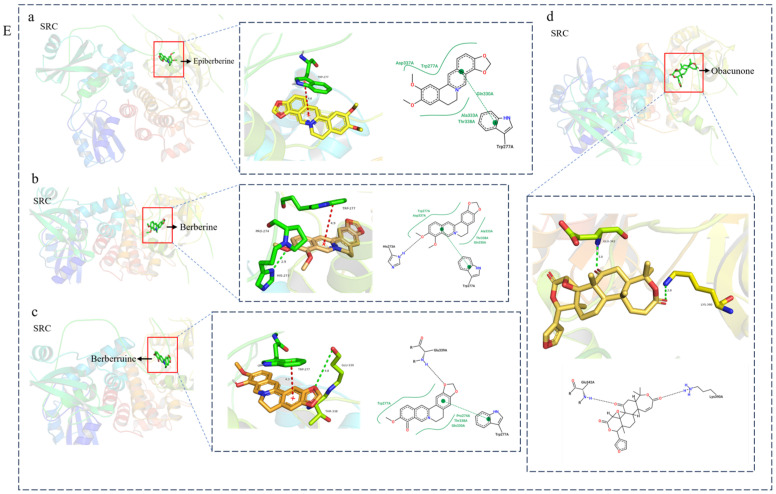
Binding energies, binding sites, and modes of action of epiberberine, palmatine, worenine, obacunone, berberine, and berberruine and receptor proteins STAT3, ESR1, CASP3, and SRC. (**A**): Heatmap of binding energies between active drugs and STAT3, ESR1, CASP3, and SRC. (**B**): Schematic diagram of interaction patterns between epiberberine and palmatine with STAT3. (**C**): Schematic diagram of interaction patterns between worenine and ESR1. (**D**): Schematic diagram of interaction patterns between obacunone and CASP3. (**E**): Schematic diagram of interaction patterns between epiberberine, berberine, berberrubine, and obacunone with SRC.

In the molecular docking test of small molecule ligands and receptor proteins, a lower binding energy indicates a more stable binding conformation of small molecule ligands and receptor proteins. In this study, the binding energies of epiberberine, palmatine, worenine, obacunone, berberine, and berberruine with the main target receptor proteins STAT3, ESR1, CASP3, and SRC were all negative, indicating that the compounds and receptors could be docked and exhibited good binding. The molecular docking verification of the main targets of small molecule epiberberine, palmatine, worenine, obacunone, berberine, and berberruine revealed that epiberberine and palmatine exhibited high binding affinity to the STAT3 protein, worenine demonstrated high binding affinity to the ESR1 protein, obacunone exhibited high binding affinity to the CASP3 protein, and epiberberine, obacunone, berberine, and berberruine exhibited high binding affinity to the SRC protein. The interaction between epiberberine and palmatine with the STAT3 protein was notable. The binding energy of the Epiberberine-STAT3 complex was −7.6. The connection position was located at the STAT3 amino acid residues Tyr446A and Lys363A, and two hydrogen bonds were generated at the docking position. The binding energy between palmatine and STAT3 was found to be 7.6. The binding site was identified at the amino acid residue Thr500A of the STAT3 protein, and a hydrogen bond was formed at the docking site. Worenine demonstrated a favorable interaction with the ESR1 protein. The binding energy was found to have an absolute value of 7.79. The binding site was identified at the amino acid residue Trp393A of the ESR1 protein, and a hydrogen bond was formed at the docking site. Obacunone demonstrated a favorable interaction with the CASP3 protein, with an absolute binding energy value of 8.31. The connection position is situated at the CASP3 protein base acid residues, namely Arg207A, Arg64A, Tyr204A, and Gln161A. Four hydrogen bonds are generated at the docking position. The compounds epiberberine, berberine, obacunone, and berberrubine demonstrated a favorable interaction with the SRC protein. The binding energy of the epiberberine-SRC protein complex was found to be 7.36 kcal/mol. The connection was established at the amino acid residue Trp277A of the SRC protein, resulting in the formation of a π bond at the docking position. The binding energy of berberine and the SRC protein was found to be 8.43. The connection position was identified at the amino acid residues Trp277A and His273A of the SRC protein, and a π bond and a hydrogen bond were generated at the docking position. The binding energy between berberrubine and the SRC protein was found to be 8.08. The binding site was identified at the amino acid residues Trp277 A and Glu339 A of the SRC protein, and a π bond and a hydrogen bond were formed at the docking site. The binding energy between obacunone and the SRC protein was found to be 9.29 kcal/mol. The connection position was identified at the amino acid residues Lys390A and Glu342A of the SRC protein, with two hydrogen bonds generated at the docking position (Figure 5).

## 4. Discussion

Network pharmacology is a discipline that employs large-scale data and computer technology to investigate the interactions between drug molecules and biological targets, pathways, genes, proteins, and other molecules in vivo. To achieve this, an interaction network between drug molecules and proteins can be constructed. By analyzing the relationships between these interactions and investigating their effects on drug efficacy, toxicity, and metabolism, this approach aids in developing efficient solutions for drug discovery, therapeutic treatments, and drug repurposing [6]. In this study, based on network pharmacology, the potential targets of *Coptidis rhizoma* against PEDV were screened using the TCMSP pharmacological database, SwissDrugDesign database, GeneCards database, and UniProt database, and 74 specific targets of *Coptidis rhizoma* against PEDV were obtained. The core gene targets were screened and the interaction network diagram was made. Finally, 14 core targets were obtained. They were STAT3, ESR1, CASP3, SRC, MMP9, EGFR, PTGS2, MDM2, ICAM1, IKBKB, HSP90AA1, PTK2, SIRT1, MAPK8, among which STAT3, ESR1, CASP3, and SRC had the greatest interactions. Among the core targets, STAT3 is a member of the STAT family and is involved in a number of biological processes, including cell proliferation, survival, differentiation, and angiogenesis. In normal cells, STAT3 primarily transmits the transcriptional signals of cytokines and growth factor receptors on the plasma membrane to the nucleus through the immediate activation of phosphorylation, thereby facilitating the exchange of signals between the cytoplasm and the nucleus for a series of biological processes [7]. It is therefore proposed that the replication of PEDV may be inhibited by targeting the STAT3 signaling pathway. This could be achieved by developing STAT3 inhibitors or degradation agents. In the data of Huang H. et al. [8], the infection of PEDV has been observed to up-regulate the protein tyrosine phosphatase non-receptor type 14 (PTPN14), a potential tumor suppressor, and to reduce the phosphorylation of STAT3 and inhibit the activation of STAT3. The study conducted by Yang J. et al. also revealed that the inhibition of the STAT3 signaling pathway could alleviate the inflammatory response and reduce intestinal damage induced by PEDV [9]. Additionally, the study conducted by Li X. et al. indicates that the overexpression of STAT3 may facilitate the replication of PEDV [10]. The aforementioned studies have demonstrated that the STAT3 signaling pathway is a pivotal factor in the pathogenesis of PEDV infection. Inhibition of the STAT3 signaling pathway results in the inhibition of PEDV infection, whereas overexpression of STAT3 promotes PEDV replication. In the study of anti-PEDV infection of *Coptidis rhizoma* active drugs, it was observed that STAT3 can interact with epiberberine and palmatine, which serve as important targets. It can be seen that the STAT3 signaling pathway plays an important role in the anti-PEDV infection of *Coptidis rhizoma* active drugs; however, the specific molecular mechanism remains to be further elucidated.

ESR1 is a critical estrogen receptor significantly associated with the development and prognosis of estrogen-dependent malignant tumors [11]. To date, there have been few reports on the relationship between ESR1 and PEDV. Studies have demonstrated that ESR1 is closely related to the NF-κB signaling pathway. The interaction between ESR1 and NF-κB is inhibitory; ESR1 reduces NF-κB DNA binding activity and suppresses NF-κB-mediated transcription of the IL6 promoter. In addition, ESR1 can replace RELA/p65 and associated co-regulators at the promoter, leading to the inhibition of NF-κB [12]. Research indicates that the cooperation between ESR1 and NF-κB activates transcription through the recruitment of adjacent response elements, thereby promoting cell signal transmission [12]. However, it remains to be determined whether the anti-PEDV effect of ESR1 is mediated by the NF-κB signaling pathway.

CASP3 is a protease that exhibits specific cleavage activity towards poly ADP ribose polymerase (PARP1) and acetyl-DEVD-7-amino-4-methylcoumarin (Ac-DEVD-AMC), resulting in DNA cleavage and the promotion of apoptosis [13]. In the data gathered by Zhou H.C. et al. [14], overexpression of cell communication network factor 1 (CCN1) was observed to increase the phosphorylation level of p53, promote the expression of Bax and the cleavage of CASP9 and CASP3, and inhibit the production of Bcl-2. The knockdown of CCN1 has been demonstrated to reduce the phosphorylation level of p53, inhibit the production of Bax and the cleavage of CASP9 and CASP3, and promote the expression of Bcl-2. In the study by XU X.G. et al. [15], it was demonstrated that the nonstructural protein Nsp9 of porcine epidemic diarrhea virus (PEDV) interacts with the H2BE protein. Overexpression of H2BE protein was found to inhibit the expression of Bax and the cleavage of CASP9 and CASP3, while promoting the expression of Bcl-2. Previous studies have demonstrated that PEDV infection can alter the CASP3 protein through various mechanisms that influence apoptosis. The primary determinant is the impact of pro-apoptotic protein Bax expression. When Bax is overexpressed, CASP3 is activated, leading to apoptosis and subsequently promoting PEDV replication.

SRC is a non-receptor tyrosine protein kinase encoded by a specific proto-oncogene, which plays a pivotal role in the growth, progression, and metastasis of cancer [16]. In the context of infection by porcine epidemic diarrhea virus (PEDV), SRC plays a pivotal role in the coordination and promotion of cell signaling pathways. The entry of PEDV into cells is primarily dependent on the endocytosis of transferrin receptor 1 (TfR1), which necessitates the regulation of SRC [17]. TfR1 binds to SRC and interacts with SRC, which increases the phosphorylation of TfR1 Tyr20 and promotes viral replication. Conversely, the use of SRC inhibitors has been demonstrated to reduce the phosphorylation of TfR1 Tyr20 and to inhibit viral replication [18]. In Li X.M.’s report [10], leflunomide active metabolite A77 1726 was also found to effectively limit PEDV replication by inhibiting JAKs and SRC. The importance of SRC in PEDV replication indicates that the study of SRC inhibitors provides a new strategy for anti-PEDV infection. Furthermore, additional potential molecular mechanisms and signaling pathways of *Coptidis rhizoma* against PEDV were identified through GO enrichment and KEGG enrichment. These include protein serine/threonine/tyrosine kinase activity, zinc ion binding, and transmembrane receptor protein tyrosine kinase activity. Additionally, the following processes are positively regulated: RNA polymerase II promoter transcription, apoptosis, protein kinase B signal, and others. The transmembrane receptor protein tyrosine kinase signal pathway, protein autophosphorylation, PI3K-Akt signal pathway, fluid shear stress and atherosclerosis, chemical carcinogen-receptor activation, human cytomegalovirus infection, microRNA in cancer, and endocrine resistance and Alzheimer’s disease were also identified. The specific action sites of the active ingredients of the drug against PEDV were identified through molecular docking testing. For instance, epiberberine and palmatine demonstrated a high affinity for the STAT3 protein, worenine exhibited a high affinity for the ESR1 protein, and obacunone exhibited a high affinity for the CASP3 protein. Epiberberine, obacunone, berberine, and berberruine demonstrated high binding affinity for the SRC protein. In conclusion, *Coptidis rhizoma*, as a pure natural drug of traditional Chinese medicine, contains a variety of effective active ingredients, which have the advantages of a wide effect, safety, low residue, low toxicity, and not easy to produce drug resistance. The anti-PEDV mechanism is multi-target, multi-mechanism, and multi-pathway, with multiple components working in concert. However, the precise body mechanism remains to be elucidated. Notably, this study used the GeneCards database, which focuses on human-related diseases, to research PEDV, but there are certain limitations. Significant genetic differences exist between humans and pigs; some genes critical to human diseases may exhibit different expressions or functions in pigs. Nevertheless, comparative genomics shows that over 80% of genes are homologous between pigs and humans, indicating a degree of biological similarity [19]. This similarity renders pigs valuable models for studying human diseases, drug development, and organ transplantation [20]. Conversely, whether human models can be utilized to study porcine diseases remains to be investigated. Additionally, PEDV, as a member of the coronavirus family, exhibits strong adaptability and extensive mutability [3]. Research indicates that PEDV can replicate in human intestinal cells, suggesting the possibility of cross-species infection, albeit weakly. This potential highlights the promise of research into cross-species coronavirus pathogenicity or host migration [21]. Hence, the feasibility of applying human disease targets to PEDV research warrants further investigation. Nevertheless, the use of the GeneCards database in this study has certain limitations, and subsequent research outcomes might not show significant antiviral effects in pig populations. However, these findings could still be crucial for other animals, including humans, in the future, thus bearing significance and value for public safety. Additionally, it is noteworthy that this study conducted extensive searches in the GeneCards database to obtain more target data.

## 5. Conclusions

This study identified six key active components of *Rhizoma coptidis* and four crucial potential targets for anti-PEDV infection through network pharmacology and molecular docking technology. The findings indicated that the active pharmaceutical ingredients of *Rhizoma coptidis* demonstrated considerable potential for the prevention and control of PEDV infection. The findings of this study provide essential background information for further investigation of the anti-PEDV infection properties of the active pharmaceutical ingredients of *Rhizoma coptidis*. Furthermore, this study has identified potential signalling pathways for some *Rhizoma coptidis* active drugs in the context of PEDV infection. Although not yet verified, this provides new avenues for further study of the molecular mechanisms of *Rhizoma coptidis* active drugs against PEDV infection, which may yield valuable insights.

## Figures and Tables

**Table 1:** Candidate compounds and corresponding targets of main active components of *Coptidis rhizoma*.

Molecule Name	Chemical Structural	Molecule ID	Molecular Weight	Oral Bioavailability (%)	Drug-Likeness	Targets
Berberine	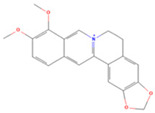	MOL001454	336.39	36.86	0.78	100
Obacunone	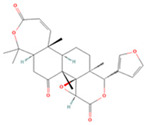	MOL013352	454.56	43.29	0.77	100
Berberrubine	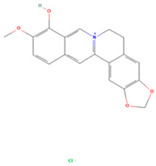	MOL002894	322.36	35.74	0.73	22
Epiberberine	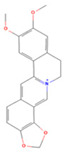	MOL002897	336.39	43.09	0.78	100
(R)-Canadine	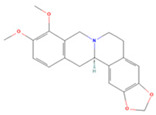	MOL002903	339.42	55.37	0.77	100
Berlambine	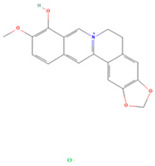	MOL002904	351.38	36.68	0.82	100
Corchoroside A_qt	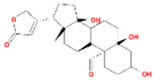	MOL002907	404.55	104.95	0.78	2
Magnograndiolide	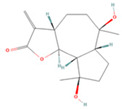	MOL000622	266.37	63.71	0.19	49
Palmidin A	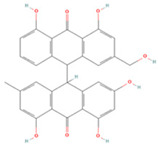	MOL000762	510.52	35.36	0.65	0
Palmatine	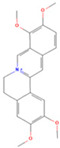	MOL000785	352.44	64.60	0.65	100
Quercetin	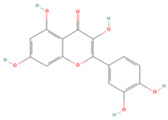	MOL000098	302.55	46.43	0.28	154
Coptisine	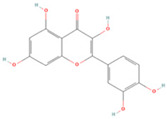	MOL001458	320.34	30.67	0.86	23
Worenine	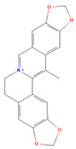	MOL002668	334.37	45.83	0.87	23
Moupinamide	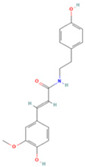	MOL008647	313.38	86.71	0.26	100

## Data Availability

All pertinent data are presented in the aforementioned paper.
